# Case Report: Rare brainstem involvement in anti-metabotropic glutamate receptor 1 encephalitis

**DOI:** 10.3389/fimmu.2026.1831181

**Published:** 2026-08-18

**Authors:** Meijiao Hu, Heng Zhou, Xin Yi

**Affiliations:** 1 Department of Neurology, People's Hospital of Xiangxi Tujia and Miao Autonomous Prefecture/The First Affiliated Hospital of Jishou University, Jishou, China; 2 Department of Radiology, People's Hospital of Xiangxi Tujia and Miao Autonomous Prefecture/The First Affiliated Hospital of Jishou University, Jishou, China

**Keywords:** anti-mGluR1 encephalitis, autoantibody, autoimmune encephalitis, brainstem involvement, case report

## Abstract

Anti–metabotropic glutamate receptor 1 (mGluR1) encephalitis is a rare form of autoimmune encephalitis that typically presents with cerebellar dysfunction. Predominant brainstem involvement has seldom been reported. We report an 87-year-old man who presented with a subacute onset of fever, dysarthria, limb weakness, and altered mental status. Comprehensive diagnostic evaluation revealed anti-mGluR1 antibodies in both serum and cerebrospinal fluid using a cell-based assay, while cerebrospinal fluid next-generation sequencing (NGS) was negative for infectious pathogens. Brain MRI demonstrated extensive T2-weighted and fluid-attenuated inversion recovery (T2/FLAIR) hyperintensity involving the midbrain, pons, and medulla, without diffusion restriction. These findings support the possibility that anti-mGluR1 encephalitis may, in rare instances, present with predominant brainstem involvement. Testing for anti-mGluR1 antibodies may be considered in patients with otherwise unexplained subacute inflammatory brainstem syndromes.

## 1 Introduction

Autoimmune encephalitis is an immune-mediated inflammatory disease caused by autoantibodies targeting neuronal surface antigens in the central nervous system. Among them, metabotropic glutamate receptor 1 (mGluR1) encephalitis is an extremely rare subtype. mGluR1 belongs to the G protein-coupled receptor family and is predominantly expressed on the dendrites of Purkinje cells in the cerebellum. It is also distributed in several other brain regions, including the olfactory bulb, thalamus, hippocampus, globus pallidus, substantia nigra, deep cerebellar nuclei, and the superior colliculus (1). When this receptor is subjected to autoimmune attack, it can trigger the rare condition of autoimmune encephalitis. Previously reported cases have primarily featured cerebellar involvement as the main clinical manifestation, although other regions such as the thalamus, occipital lobe, frontal lobe, parietal lobe, and spinal cord can also be affected (2–5). We report an unusual case of anti-mGluR1 encephalitis characterized by predominant brainstem involvement, a presentation that has rarely been described in the literature.

## 2 Case presentation

The patient is an 87-year-old male who presented to our hospital with a 9-day history of progressive weakness in the limbs, dysarthria, and urinary/fecal incontinence. Three days prior to admission, he developed fever, cough with expectoration, confusion and delirium. He had a history of hypertension but no other significant medical history. He was taking antihypertensive medications regularly, but denied any other medications or family history of disease. On admission, his temperature was 39.0 °C, pulse rate 90 beats/min, respiratory rate 25 breaths/min, and blood pressure 143/63 mmHg. He was disoriented and had slurred speech. Bilateral lung auscultation revealed coarse breath sounds. Muscle strength in the lower limbs was graded 3/5, while upper limb strength was 4/5. The left lower limb exhibited hyperreflexia, but no pathological signs were elicited. Initial imaging included a CT scan of the brain, revealing multiple lacunar infarcts in the bilateral basal ganglia, white matter atrophy, and cerebral atrophy. Chest CT showed no significant abnormalities. Laboratory results showed a C-reactive protein level of 49.82 mg/L (normal range: 0–9 mg/L). Procalcitonin was 0.18 ng/mL (normal range: 0-0.05 ng/mL). B-type natriuretic peptide (BNP), liver and renal function tests, hepatitis B surface antigen, HIV antibodies, syphilis antibodies, and thyroid function were within normal limits. Testing for influenza A/B, SARS-CoV-2, and other respiratory pathogens was negative. Screening for serum tumor markers—including alpha-fetoprotein (AFP), carcinoembryonic antigen (CEA), total prostate-specific antigen (tPSA), and free prostate-specific antigen (fPSA)—was completed, all of which yielded negative results. The initial differential diagnosis included intracranial infection and acute cerebrovascular disease. Empirical treatment was initiated with intravenous acyclovir and meropenem. Aspirin and atorvastatin were administered for secondary prevention of suspected cerebrovascular disease. On the third day of hospitalization, a lumbar puncture was performed, revealing a cerebrospinal fluid pressure of 115 mmH2O and elevated protein levels at 1210.9 mg/L (normal range: 150–450 mg/L). The CSF cell count, white blood cell count, glucose, chloride, C-reactive protein, adenosine deaminase, and lactate dehydrogenase were all normal. Gram stain, acid-fast stain, and India ink stain did not show abnormalities. Later that evening, the patient developed respiratory distress with pink frothy sputum, prompting further analysis. Blood gas analysis indicated type I respiratory failure. BNP was markedly elevated at 5665 pg/mL (normal range:0–900 pg/mL). A 15-lead electrocardiogram demonstrated findings consistent with an acute anterior myocardial infarction. A bedside chest X-ray suggested a lower lung infection. Due to the patient’s advanced age and worsening condition, he was transferred to the ICU for further management.

**Figure 1:**
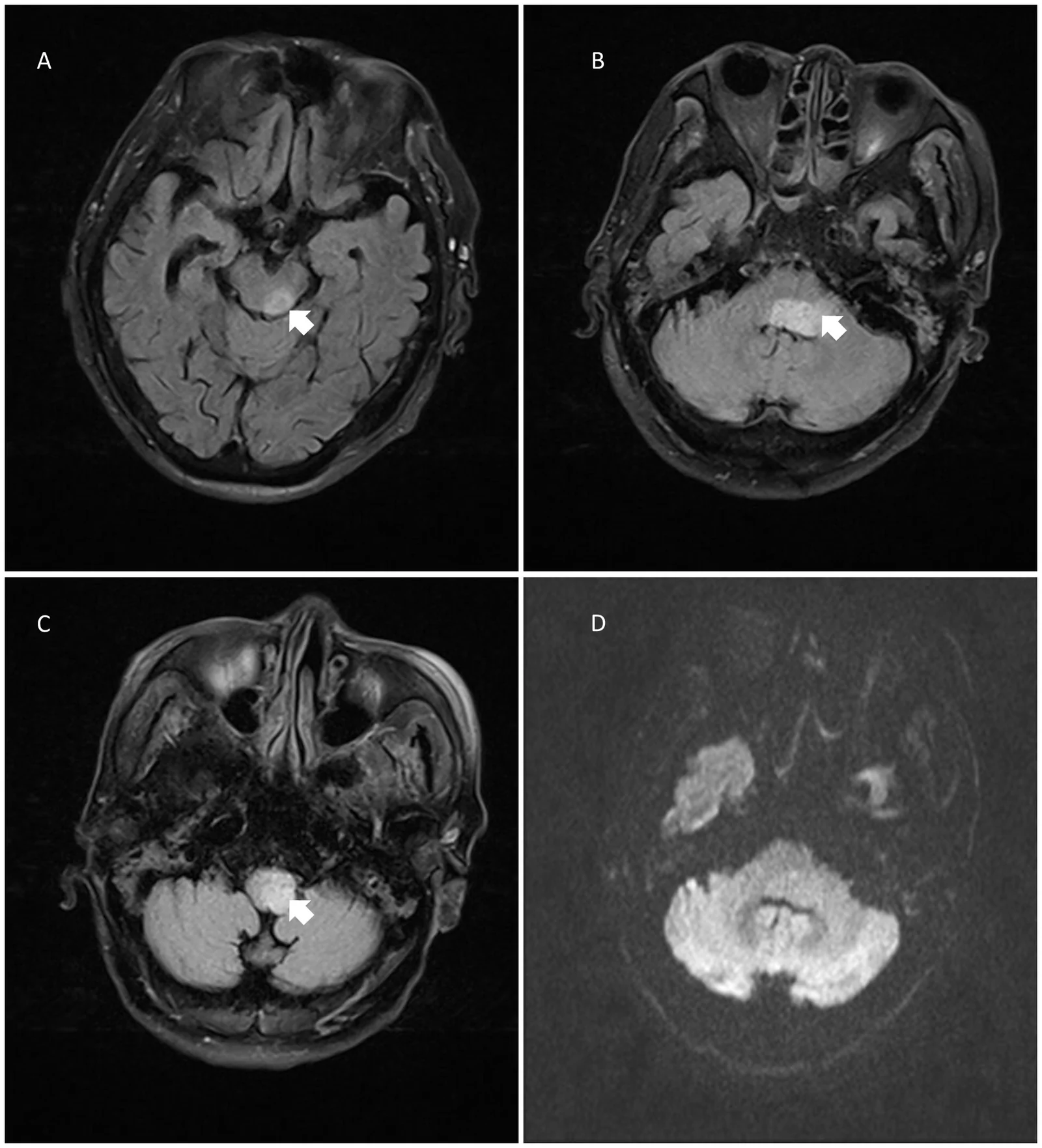
MRI of the brain showed brainstem involvement, including the left midbrain **(A)**; arrowhead), pons **(B)**; arrowhead), and the majority of the medulla oblongata **(C)**; arrowhead), with hyperintensity on FLAIR. DWI **(D)** showed no evidence of diffusion restriction.

On the 7th day of hospitalization, CSF NGS was normal. Anti-mGluR1 antibodies were detected in both serum and CSF using a commercially available cell-based assay (CBA) performed at KingMed Diagnostics Center. Antibodies against other glutamate receptors, including anti-NMDA receptor, anti-AMPA1, anti-AMPA2, anti-LGI1, anti-CASPR2, anti-GABAB, anti-IgLON5, anti-DPPX, anti-GlyR1, anti-DRD2, anti-GAD65, anti-mGluR5, anti-Neurexin-3a, anti-GABAA, anti-KLHL11, anti-ganglionic AChR, anti-AQP4, anti-MOG, and anti-GFAP, were all negative. Brain MRI revealed extensive, contiguous high signal intensity areas on T2-weighted and FLAIR sequences, primarily affecting the left brainstem. The involvement extended from the midbrain to the pons and medulla oblongata, with slight extension into the right brainstem (**Figure 1**). These lesions showed an asymmetric distribution, predominantly on the left side, without obvious mass effect or hemorrhagic changes. Diffusion-weighted imaging (DWI) showed no diffusion restriction, and no corresponding low signal was seen on the apparent diffusion coefficient (ADC) map. Magnetic resonance angiography (MRA) findings were unremarkable. Abdominal and urinary tract ultrasound did not reveal any suspicious malignant lesions. Contrast-enhanced MRI and PET-CT were not conducted, as the patient developed rapid clinical deterioration and respiratory failure, and could not tolerate lengthy diagnostic procedures. Based on the subacute onset symptoms, cerebrospinal fluid findings, imaging results, laboratory tests, and clinical examination, and exclusion of alternative infectious and autoimmune etiologies, a diagnosis of anti-mGluR1 encephalitis was considered. Based on current recommendations for autoimmune encephalitis, the patient was treated with high-dose intravenous methylprednisolone and intravenous immunoglobulin. Given the severity of neurological involvement, mycophenolate mofetil(MMF) was added as a steroid-sparing immunosuppressive agent. Despite treatment, no significant neurological improvement was observed. Unfortunately, the clinical course was further complicated by the patient’s advanced age; he developed an acute extensive anterior myocardial infarction, which was subsequently exacerbated by a bilateral lower lung infection. The patient passed away on the 28th day of hospitalization due to respiratory and circulatory failure. The patient's clinical course, including symptom onset, diagnostic evaluation, treatment, and clinical outcome, is summarized in **Table 1**.

**Table 1:** Chronological timeline of clinical events.

Time	Clinical events
Day -9	Progressive limb weakness, dysarthria, urinary and fecal incontinence
Day -3	Fever, cough, confusion, delirium
Day 1	Hospital admission; initial diagnosis, assessment, empirical acyclovir, meropenem, aspirin, atorvastatin initiated
Day 3	Lumbar puncture performed; CSF protein elevated
Day 3	Respiratory failure, acute myocardial infarction, ICU admission
Day 7	CSF NGS negative
Day 7	Serum and CSF anti-mGluR1 antibodies positive
Day 7	MRI revealed extensive brainstem lesions
Day 7	IVIG and methylprednisolone pulse therapy initiated; MMF added
Day 7–28	No significant neurological improvement
Day 28	Death due to respiratory and circulatory failure

## 3 Discussion

### 3.1 Clinical significance of brainstem involvement

The diagnosis of anti-mGluR1 antibody-associated encephalitis is highly challenging due to the heterogeneity of its clinical manifestations and auxiliary test results. While the majority of reported cases in the literature describe adults with a subacute onset characterized predominantly by cerebellar involvement, our patient exhibited a rare brainstem-predominant clinicoradiological phenotype. Although a single case cannot establish causality, this observation adds to the limited literature indicating that anti-mGluR1 encephalitis may sometimes present primarily with brainstem involvement. Clinicians should therefore consider anti-mGluR1 encephalitis in the differential diagnosis of patients with unexplained subacute inflammatory brainstem syndrome, especially after excluding conventional infectious, vascular, and autoimmune causes.

### 3.2 Differential diagnosis

Patients with subacute brainstem inflammatory syndrome, particularly elderly individuals, require a meticulous evaluation of a broad differential diagnosis. Although the detection of anti-mGluR1 antibodies in both serum and cerebrospinal fluid supported an autoimmune etiology, other possible diagnoses were systematically considered.

CLIPPERS: Chronic lymphocytic inflammation with pontine perivascular enhancement responsive to steroids (CLIPPERS) predominantly affects the brainstem and may present with overlapping clinical features such as ataxia, dysarthria, and cranial nerve deficits. Radiologically, CLIPPERS is characterized by a “peppering” pattern on contrast-enhanced MRI, consisting of multiple punctate or curvilinear gadolinium-enhancing lesions primarily involving the pons and adjacent structures (6, 7). Recent case reports have further emphasized that CLIPPERS typically demonstrates punctate gadolinium-enhancing lesions and favorable responses to corticosteroid-based immunotherapy, even in severe presentations (8). In our patient, a contrast-enhanced MRI could not be performed due to critical illness and intolerance for prolonged imaging. Non-contrast MRI demonstrated confluent T2/FLAIR hyperintensities in the midbrain, pons, and medulla, lacking the punctate distribution typical of CLIPPERS. Moreover, CLIPPERS usually shows rapid improvement with corticosteroids, whereas our patient exhibited steroid-resistant progressive decline despite high-dose methylprednisolone, intravenous immunoglobulin, and adjunctive mycophenolate mofetil. Anti-mGluR1 antibodies were detected in both serum and cerebrospinal fluid. Combined with the clinical and imaging findings, this evidence further supports a diagnosis of anti-mGluR1 encephalitis rather than CLIPPERS syndrome.

Primary CNS Tumors: Primary central nervous system (CNS) tumors remain an important differential diagnosis in elderly patients with progressive brainstem lesions. Epidemiological studies indicate that gliomas are among the most common primary CNS tumors in adults (9, 10), although adult brainstem gliomas are rare, accounting for less than 2% of adult gliomas (11). Brainstem gliomas typically demonstrate infiltrative growth, focal enlargement, and T2/FLAIR hyperintensity on MRI, while high-grade lesions may additionally exhibit mass effect, necrosis, or hemorrhagic change (11, 12). Primary CNS lymphoma (PCNSL), although rare in the brainstem, is characterized by high cellularity and relatively homogeneous lesions with marked diffusion restriction on DWI and corresponding low ADC values (13). Although contrast-enhanced MRI and histopathological confirmation were unavailable, which precluded the definitive exclusion of neoplastic disease, the overall clinical, radiological, and immunological findings were considered more consistent with anti-mGluR1 encephalitis rather than a brainstem glioma or PCNSL.

Other Autoimmune/Inflammatory Disorders: Glial fibrillary acidic protein (GFAP) astrocytopathy, myelin oligodendrocyte glycoprotein antibody-associated disease (MOGAD), aquaporin-4-related neuromyelitis optica spectrum disorder (AQP4-NMOSD), and paraneoplastic encephalitis were also considered. Testing for GFAP, MOG, and AQP4 antibodies was negative, and neuroradiological findings were inconsistent with these disorders. Paraneoplastic encephalitis remained a consideration given the patient’s age and the known association between anti-mGluR1 antibodies and malignancy. No clinical evidence of malignancy was found in the current evaluation. However, since a comprehensive oncological workup—including PET-CT—was not performed, the possibility of an occult tumor cannot be entirely excluded.

In conclusion, despite limitations such as the absence of contrast-enhanced MRI and incomplete oncological evaluation, the combination of subacute brainstem syndrome, diffuse T2/FLAIR hyperintensity without diffusion restriction, negative infectious workup, and concordant antibody positivity is most consistent with anti-mGluR1 encephalitis.

### 3.3 Pathogenesis and paraneoplastic context

The exact pathogenesis of anti-mGluR1 encephalitis remains to be fully elucidated, but it may be associated with paraneoplastic syndromes, infections, and systemic autoimmune diseases. Literature reports indicate that some cases are linked to malignancies such as lymphoma, leukemia, prostate cancer, and testicular seminoma, suggesting a paraneoplastic etiology (4, 14, 15). Additionally, studies have suggested that infections, including herpes zoster, streptococcal infections, and dengue virus, may act as potential triggering factors (4, 5, 16). Furthermore, some cases have been associated with autoimmune diseases, such as Sjögren’s syndrome, pernicious anemia, and Hashimoto’s thyroiditis (4), further supporting the hypothesis that immune dysregulation contributes to disease development. In our patient, a comprehensive oncological screening-including a PET-CT evaluation-was precluded by his rapidly deteriorating neurological status and respiratory failure. Consequently, an underlying occult neoplasm cannot be definitively excluded.

### 3.4 Biological plausibility of brainstem involvement

The rarity of this case lies in its predominant and extensive brainstem involvement, a phenotype seldom reported in anti-mGluR1 encephalitis. The mGluR1 receptor is well known for its high-density expression and critical function in Purkinje cells of the cerebellum. Available anatomical and molecular evidence suggests that mGluR1 is also expressed in selected brainstem nuclei. Classic *in situ* hybridization studies have shown that mGluR1 mRNA can be detected in several regions of the central nervous system, including specific brainstem nuclei, indicating that its distribution is not limited to the cerebellum (1). Additionally, studies have demonstrated that mGluR1 receptors are expressed in brainstem structures associated with auditory processing, such as the cochlear nucleus, inferior colliculus, and other relay nuclei (17). These anatomical findings suggest that although mGluR1 is expressed at much lower levels in the brainstem than in the cerebellum, its presence in specific brainstem nuclei offers a potential anatomical basis for the observed brainstem-predominant phenotype. Taken together, these anatomical observations provide a potential biological rationale for the hypothesis that anti-mGluR1 encephalitis may occasionally present with a brainstem-predominant phenotype. However, this case does not establish a causal link between regional mGluR1 expression and selective brainstem involvement. Further experimental and clinicopathological studies are needed to clarify this association.

### 3.5 Treatment and prognosis

The detection of anti-mGluR1 antibodies in both serum and cerebrospinal fluid provides important diagnostic support when interpreted in the appropriate clinical context. In this case, both cerebrospinal fluid and serum tested positive for anti-mGluR1 antibodies. According to current expert consensus (18), anti-mGluR1 antibodies are rare and highly specific; concordant positivity in both serum and CSF is considered diagnostically meaningful in an appropriate clinical context. Once the diagnosis is confirmed by clinical, imaging, and immunological findings, immunotherapy is initiated. Currently, there is no standardized treatment protocol for this disease. First-line immunotherapy includes high-dose corticosteroids, intravenous immunoglobulin (IVIG), and/or plasma exchange, which are often associated with clinical improvement when initiated early. Second-line therapies, including rituximab, cyclophosphamide, azathioprine, or mycophenolate mofetil, may be considered for incomplete response or relapse (19). In this case, MMF was introduced early as a steroid-sparing maintenance immunosuppressant due to its delayed onset of action and the expected need for long-term immunosuppression if the patient responded to first-line therapy. Although rituximab is an alternative second-line option, escalation to rituximab was deemed unfeasible because the patient’s condition rapidly deteriorated with acute anterior myocardial infarction, respiratory failure, and severe pulmonary infection. Under these circumstances, the potential risks of B-cell-depleting therapy were considered to outweigh the benefits. A poor prognosis may be associated with severe neurological involvement, structural MRI abnormalities, delayed or refractory disease course, and systemic complications (19). In our patient, despite first-line immunotherapy and adjunctive MMF, no significant neurological improvement was observed. The fatal outcome was likely multifactorial, resulting from the combined impact of extensive brainstem involvement, advanced age, acute anterior myocardial infarction, respiratory failure, and severe pulmonary infection, rather than being solely attributable to anti-mGluR1 encephalitis.

### 3.6 Limitations

Several limitations should be acknowledged. First, contrast-enhanced MRI and comprehensive oncological evaluation, including PET-CT, could not be performed due to the patient’s clinical instability and poor tolerance for prolonged imaging. Second, serial antibody testing and follow-up imaging were unavailable, precluding assessment of the relationship between antibody dynamics, radiological evolution, and treatment response. Third, histopathological confirmation was lacking. Finally, as this is a single case report, no causal relationship between anti-mGluR1 autoimmunity and the brainstem-predominant phenotype can be established. These limitations hinder the assessment of disease progression and treatment response, prevent the complete exclusion of alternative causes, and highlight the need for further clinicopathological studies.

## 4 Conclusion

In conclusion, this case suggests a possible association between anti-mGluR1 antibody-mediated autoimmunity and extensive brainstem-predominant inflammation, thereby modestly expanding the recognized clinical and neuroradiological spectrum of the disease. Our findings support the hypothesis that selective brainstem involvement may represent an uncommon phenotype of anti-mGluR1 encephalitis. Further studies are needed to clarify the relationship between anti-mGluR1 autoimmunity and selective brainstem involvement. Therefore, in patients presenting with unexplained subacute inflammatory brainstem syndromes, clinicians should consider incorporating anti-mGluR1 antibody testing into the comprehensive diagnostic workup, even in the absence of classic cerebellar involvement.

## Author’s note

The views expressed in this article are those of the authors and do not necessarily reflect the official policy or position of their affiliated institutions.

## Data Availability

The raw data supporting the conclusions of this article will be made available by the authors, without undue reservation.
